# Update on Laser Trabeculoplasty

**DOI:** 10.4103/0974-9233.56221

**Published:** 2009

**Authors:** Fathi El Sayyad, Magdi Helal

**Affiliations:** Misr University for Science and Technology, Executive Director, ElSayyad Eye Center, Cairo, Egypt; 1 Chief Glaucoma Service, Magrabi Eye Hospital, Cairo, Egypt

**Keywords:** Laser, trabeculoplasty, update

## Abstract

Newer techniques of Laser Trabeculoplasty have revived the procedure and gained widespread acceptance by the ophthalmic community. This review was undertaken to address the evolution of different laser trabeculoplaty techniques, proposed mechanisms of action as well as review current studies of the therapeutic effects of these interventions.

## INTRODUCTION AND HISTORICAL REVIEW

Laser trabeculoplasty (LT), first described in 1974 by Worthen and Whickham,^1^ became a commonly performed procedure for the treatment of primary open-angle glaucoma (POAG) after the publication of Wise and Witter's^2^ pilot study in 1979 - LT with argon lasers (argon laser trabeculoplasty; ALT).

Several other lasers including krypton (647.1 or 568.2 nm) diode (810 nm), DLT and continuous wave, frequency-doubled Nd: YAG (532 nm), the work by Latina and colleagues (1998)^3^ directly led to selective laser system (SLT) used in clinical practice today. The commercially available SLT laser system is a Q-switched, frequency-doubled, 532-nm Nd: YAG laser. Micro pulse Diode Laser Trabeculoplasty MLT utilizes the micro pulse 810-nm diode LT (Micro pulse-Diode LT or MLT)^4^

## INDICATIONS

Most glaucoma specialists regard LT as an adjunct to maximum tolerated medical therapy. Alternatively, it can be used as an initial glaucoma therapy. LT provides successful results in POAG, pseudo exfoliation and pigmentary glaucoma and in eyes with combined mechanism glaucoma. It does not appear to be effective in glaucoma secondary to uveitis, congenital glaucoma, juvenile open angle glaucoma, iridocorneal endothelial syndrome, iridocorneal mesodennal dysgenesis, steroid induced glaucoma, and glaucoma secondary to elevated episcleral venous pressure.

## NEW TECHNIQUES

SLT results in selective absorption of energy by trabecular pigmented cells sparing adjacent cells and tissues from thermal damage. It delivers a 400 um diameter treatment spot in three nanoseconds, by using short bursts at low power settings (typically in the range of 0.4 to 1.2 mJ per treatment spot for most eyes). SLT delivers energy fluence levels to the TM cells that are several thousand times lower than with ALT. One consequence of this energy reduction is that SLT seems to produce less thermal tissue damage to TM than ALT.^5^,^6^ This observation has led to significant speculation that SLT may be more repeatable than ALT.

MLT^7^ uses a large spot, low irradiance and photocoagulation protocol for the reduction of IOP with a nondestructive laser-tissue interaction to minimize collateral cell damage.

MLT utilizes an 810-nm diode laser emitting a train of repetitive short pulses to allow the surgeon to control and spatially confine the laser-induced thermal elevation to produce the intended sub lethal photo thermal effects to elicit a thermal stress response in the TM cells. It is typically performed to deliver 60 to 65 (or 120-130) confluent 300-um diameter invisible laser applications covering the whole height of the TM over 180- (or 360-) angle. Due to the combination of (a) low absorption of the 810-nm laser wavelength by the TM and (b) low irradiance of 2W over the relatively large 300 um spot, MLT seems to interact and thermally affect all superficial and deep TM-pigmented cells without producing visible photo thermal changes, tissue blanching, or bubble formation. The treatment is invisible to the surgeon and uneventful for the patient with no pain and no dazzling laser flashes (810 nm is invisible).

## RESULTS

Many prospective and retrospective studies compare SLT with ALT indicating that a similar short- and long-term efficacy of ALT and SLT can be expected in patients with OAG with a similar and a mild risk profile.^8^–^12^ Further analysis of the results shows that different percentages of intraocular pressure (IOP) reduction have been reported suggesting that the patient's selection rather than different mechanism of action is responsible for the IOP-lowering effect

The GLT results demonstrate that ALT could be considered a primary therapy for OAG. Several other studies have also demonstrated that ALT is effective at lowering IOP.^14^–^18^

Studies investigating the use of SLT as initial therapy using a 180-treatment report a mean reduction of 7.7 plus 3.5 mmHg (30%)^18^ and 8.3 mm Hg (31%).^19^

McIlraith *et al*.^19^ compare the IOP-lowering effect of SLT versus latanoprost. Investigators demonstrated an average IOP reduction of 8.3mmHg in the SLT group compared with 7.7mmHg in the medication group. There is no significant difference in pressure between the two groups over the first year of follow up. Lai *et al*.^20^ performed a small randomized trial on Chinese patients with the newly diagnosed POAG and found no significant difference in IOP control between the two groups. Overall, SLT effectiveness is similar to that of a single potent topical anti glaucoma medication with the added benefit of easier compliance. Damji *et al*.^21^ demonstrate similar IOP reduction level, at 18 months, in OAG glaucoma patients who received ALT and those who received SLT in a randomized clinical trial. The rate of failure at one year was 32% for the SLT group and 36% for the ALT group. Additional analysis of data collected from this study shows that the single factor predicting success in the SLT group is baseline IOP.

Other variables such as age, sex, type of OAG and degree of trabecular meshwork pigmentation are not associated with improved outcomes in the group of participants receiving SLT.^22^ Popiela *et al*.^23^ noted no statistically significant difference between ALT in one eye and SLT in the second eye of 27 OAG patients. The ALT group experienced a 2.6mmHg IOP decrease compared with a 2.8mmHg decrease in the SLT group. Juzych *et al.*^24^ compared the five-year outcomes of 154 patients who received ALT with 41 who received SLT. These investigators found both lasers equally effective at lowering IOP. Martinez *et al*.^25^ compared postoperative pain and anterior chamber inflammation in a prospective trial of 20 patients who received 180 degrees of ALT and 20 patients who received 180 degrees of SLT. They found no difference in pressure lowering effect between the two groups - 20% in ALT and 22% in SLT. However, the group which received ALT reported more pain and anterior chamber inflammation compared with the SLT group.

Repeat ALT effectiveness was assessed in a few studies. Feldman *et al*.^26^ found a 35% success rate for repeat ALT at six months; after one year the success rate dropped to 21% and at two years it was only 11%. Richter *et al*.^27^ found a 33% success rate in one year while Weber *et al*.^28^ and Grayson *et al*.^29^ had more favorable outcomes with repeat ALT reporting 70 and 73% success rate respectively at one year. Repeated ALT beyond 360 degrees is generally not recommended due to the risk of subsequent pressure elevation and relatively low efficacy.

Since SLT does not induce trabecular meshwork scarring, Latina *et al*.^3^ performed SLT in 23 patients who had previously undergone ALT; a 24% reduction of IOP was seen. Almost the same results were demonstrated by Kano *et al*^30^ Damji *et al.*^8^

Repeat SLT was assessed by Shah *et al*.^31^ who found 70% success rate at one year and 53% in two. Bryan *et al*.^32^ reported Reduction in IOP after SLT1 and SLT2 was significantly less with repeat treatment at one to three months, with average decreases of 5.0 and 2.9mm Hg, respectively (P is equal to 0.01); success rates for SLT1 and SLT2 were not significantly different. There was also no significant difference in eyes that received SLT2 six to 12 months after SLT1 compared with those that received SLT2 12 months or more after SLT1

The findings are encouraging and suggest that 360-degree SLT may be repeated after an initially successful 360-degree SLT fails and with IOP reduction that suggests only slightly diminishing returns. Findings also suggest that these results may be achieved as early as six months after the first treatment.

In a randomized pilot study conducted in the University of Missouri Kansas city,^7^ MLT and ALT show an equal IOP lowering effect at three months. IOP reduction from baseline is statistically significant for both study arms At one hour after treatment cells and flare reaction at trace, 1+ level was found in 10/11 (91%) of ALT eyes and 2/10 (20%) of MLT eyes.

## CONCLUSION

Along with medications, LT effectively and safely lowers elevated IOP. SLT and MLT can be performed with minimum iatrogenic damage to the TM- lowest intraoperative and postoperative complications and side-effects. This will eventually gain widespread acceptance by the ophthalmic community. Newer techniques may assume an increasingly important role of LT in our management of glaucoma.
